# Development impacts of migration and remittances on migrant-sending communities: Evidence from Ethiopia

**DOI:** 10.1371/journal.pone.0210034

**Published:** 2019-02-06

**Authors:** Misgina Asmelash Redehegn, Dingqiang Sun, Aseres Mamo Eshete, Castro N. Gichuki

**Affiliations:** 1 Department of Agricultural Economics and Management, College of Economics and Management, Nanjing Agricultural University, Nanjing, Jiangsu, China; 2 Department of Cooperative Studies, College of Business and Economics, Aksum University, Aksum, Tigray, Ethiopia; Kansas State University, UNITED STATES

## Abstract

This paper evaluates the development impacts of migration and remittances in migrant source communities by applying insights from the New Economics of Labor Migration (NELM) theory to Ethiopia’s migration. Using household survey data, we empirically evaluate how household participation in migration arises and so that the subsequent labor losses and the influx of remittances affect income sources and asset accumulation of smallholder farm households. To account several econometric issues and consistently estimate the impacts of migration and remittances, we adopted three-stage least-squares method complemented with endogeneity and multicollinearity test. Besides, using logistic and multinomial logistic regressions respectively, we estimate the determinants of the household migration decision to have migrants, as well as the probability of the household to send out temporary or permanent migrants. Findings suggest that larger and wealthier households are less likely to have migrant family members, while households living below the poverty line, as well as villages with the highest unemployment rate, are the most likely to have both temporary and permanent migrants. However, a rise in months spent out of agriculture has a significant negative effect on crop income and asset accumulation, but only for permanent migration. By contrast, the influx of remitted income from migrants has led to increased crop income and asset values in the form of land and livestock holdings. Finally, this manuscript provides more comprehensive evidence by showing the net-returns of migration in terms of initial lost-labor effects and the positive developmental impacts that it produces varied for households with different types of migration and production conditions.

## Introduction

Due to economic and political transitions, migration out of agriculture has long been a salient feature of life in rural Ethiopia. The factors influencing the household’s migration decision and so that the development impacts migration has on source communities is however quite complex and debatable following the advent of a large scale of economic and non-economic precipitates [1–3]. The decision to migrate is rational and made at the household level aimed to diversify income streams through various means in light of the fact that economic institutions do not exist to provide insurance or investment loans [4–6].

With incomplete labor markets, however, rural out-migration threatens the capacity of the household to respond to labor demand leads to the decline of agricultural production levels [7–9]. The Ethiopian agriculture would be more responsive to a reduction in available farm labor as it was featured as a traditional labor-intensive production. Further, we argue that the negative lost-labor effect is not likely to be equal for temporary and permanent migration since temporary migration implies a regular return to home to help household members to work on their farms.

By contrast, migration may reshape migrant-sending economies through indirect channels to make agricultural improvements in rural areas. Migrants often remit substantial amounts of their income share to origin families. Remittances provide the potential to foster agricultural production levels, rises household income, and improve general rural conditions [1, 10, 11]. Particularly, as rural farmers in Ethiopia live in economies virtually deficient of formal credit and insurance markets, migration may positively influence local production intensities in migrant-sending areas by providing households with scarce capital and serving as insurance policies against risks associated with new production activities [12–15].

This article aims to understand the development impacts of migration and remittances in migrant source communities by applying insights from the NELM theory to Ethiopia’s migration. The determinant factors influencing the household decision to have migrants and the selective nature of migration processes (for instance temporary or permanent migration) may have instantaneous implications on economic activities in the communities left behind. Temporary migration usually implies that it is a short-term move that allows migrants to return home, and induce productivity gains to help origin families, while permanent migration denies valuable labor for extended periods. Both temporary and permanent migration choices have sharply different net-returns, in terms of initial costs and remittances sent back home [7, 16, 17]. The nexus between the cost of losing labor to foreign labor markets and the positive economic impacts that it produces will have typical and interesting implications in low-income country settings.

Yet despite the evident importance of understanding the determinants and impacts of migration in source communities, the existing literature on Ethiopian migration has focused mostly on internal migration, urbanization and macroeconomic issues [18–20]. Few studies have explicitly addressed the issue of migration and income sources and asset accumulation in rural Ethiopia [21, 22]. Moreover, previous studies do not consider the typical economic and production conditions under which the determinants and impacts of migration and remittances occur. We further argue that the net effect of migration may vary depends on the household’s migration decision and initial asset holdings. This manuscript contributes to the existing economic literature on NELM theory by providing new empirical evidence on the determinants and developmental impacts of labor out-migration in a transition country with a predominately agrarian economy.

The rest of the text is organized as follows: in the next section, we describe the linkage of migration, remittances, and the rural economy in Ethiopia. Then the econometric methods used in the empirical study are specified. In this section, we also address the data, econometric issues and methods used to estimate the parameters. The paper presented the main empirical results with a discussion of policy implications. The final section concludes the manuscript.

## Migration, remittances and rural economy in Ethiopia

In contrast to the early 1990s, when the severe civil war pushed numerous Ethiopians outward, recently migration has increased as farmers in the rural areas struggle to reconcile livelihood degradation and extreme rural poverty coupled with oppressive political conditions [23–25]. These factors continue to adversely affecting the livelihoods of the poor farmers who mainly depend on rain-fed agriculture. Ethiopia has also a long history of poor agricultural infrastructure, inadequate risk mitigation and coping strategies and perhaps poor political will [26, 27].

Ethiopia, a hub for outward and inward migration, is one of the major migrant-sending countries and the largest-refugee hosting country in Africa [23]. Nevertheless, the country has a relatively low overall international migration rate compared to other African countries, in 2015 about two million Ethiopian migrants, accounting 2.07% of the total population, have resided outside their country. The data from the Ethiopian Ministry of Labor and Social Affairs (EMLSA) also indicate that about 460,000 Ethiopians move away from their country between 2008 and 2013. Internal or circular migration in Ethiopia is also thought to be larger compared to external flows, as households with internal migrants are estimated to be 5% of the total population [23].

**Fig 1:**
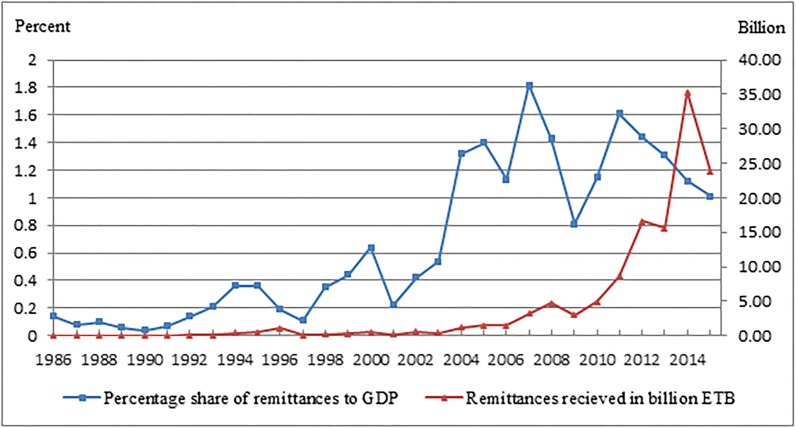
Received remittances and its percentage share to GDP for Ethiopia, 1986–2015. Source: World Bank Categories > International Data > Countries > Ethiopia.

Despite the growing rate of out-migration, origin families are still tied closely with their migrant communities and perhaps migrant return for public holidays is habitual in Ethiopia. As a result, international monetary flows to Ethiopia have increased radically. According to the World Bank report in 2015 remittance flows to Ethiopia have reached 23.75 billion Ethiopian Birr (up 20.9% over 2010). Despite the recent declines, in the last two decades, the share of remittances to GDP has increased by nearly 35.6% from 0.36% in 1995 to 1.01% in 2015 (Fig 1). The influx of these remittances into the migrant-sending areas have stimulated households to make investments in high-return activities and in fact, remittances have long been part of the risk-spreading strategies against crop shocks [6].

After experiencing economic stagnation for most of the 1970s and 1980s, Ethiopia is now one of the fastest growing economies in the world with an average growth rate of 10% annually for more than a decade. The economic growth has been driven by sustained progress in the agricultural sector, accounting for 46% of the national GDP, 83.9% of exports and remains a primary source of employment for about 80% of the labor force [28, 29]. However, as the second most populous country in Africa with an estimated population of 106 million people in 2017 the future development of agriculture is challenged by increasing small plots of farmland where farmers must earn their subsistence. The average land size in Ethiopia is low, at 0.96 hectares, and related closely to the rapid population growth [30].

In addition, the availability and access to credit and modern inputs remain one of the major challenges in rural areas of developing countries [31], and Ethiopia is no exception. Recent studies in the survey areas reveal that 60 to 80% of rural farmers are credit constrained indicated that the credit markets are thin or missing in the majority part of the country [32]. Further, many farmers in rural areas of the country do not have or have limited access to insurance markets [33]. To cope with the incidence of extreme rural poverty coupled with a desire to loosen production constraints [3, 4, 26], rural households in Ethiopia have therefore experienced widespread migration and remittances [34].

In this day, migration is transforming the socioeconomic structures of the rural economy in Ethiopia. In fact, non-migrant households are not homogenous and include households who are primarily farming and households who had sent at least one family member to work in local off-farm activities. While nearly all the households in the survey area are employed in farming activities, about 35.47% of them had at least one migrant and 18.62% of them had at least one family member working in local farm wages, and the remaining 45.91% of them are primarily farming Table 1.

**Table 1:** Migration characteristics across sample regional states.

Variable mean	Obs.	Total (n = 795)	Tigray (n = 266)	Amhara (n = 529)
% of households with migrants (all migration)	795	35.47	30.08	38.19
% of households with temporary migrants	282	15.47	12.41	17.01
% of households with permanent migrants	282	18.87	16.54	20.04
% of households with local off-farm work	795	18.62	17.29	19.28
% of primarily farm operating households	795	45.91	52.63	42.53
Average remittances sent by temporary migrants	123	48054.13	46325.30	49836.29
Average remittances sent by permanent migrants	150	83876.60	81233.12	86538.40
Average remittances received from all migrants, total	282	65965.04	65529.50	66137.03

Source: Authors’ survey.

Note: Means in this table are estimated at the individual level: for instance shares of permanent and temporary migrants and the remittances received from both migrants do not sum up to the total share of households with migrants and total remittances received by the household respectively, because there are nine households with both permanent and temporary migrants. All values are in Ethiopian Birr (ETB) (1 ETB = 0.046 US$).

Several academic evidence argue that migrant remittances support household income and remittance receiving households generally have higher levels of productivity and lower incidences of poverty than households with no remittances [35, 36]. As the size of monetary transfers became evident, remittances enable households to overcome capital constraints in their ability to achieve commercial production system [4, 37]. Remittances can also potentially contribute to the building of productive assets, such as land and livestock holdings. Furthermore, remittances are positively linked with self-employed activities and allow origin households to own rural business [38].

In our case, migrant households are found to be more productive and wealthier in terms of total income and land ownership. The average total household income is considerably higher for households that migrants would later leave, while households with no migrants appear to be poorer than the average reported. However, the share of crop income appears to be lower for households with both temporary and permanent migrants. The loss of labor might have a direct effect on crop income, but this effect seems to be moderated by the transfer of money, as 86.2% of the migrant-sending households had received average remittances of 65,965.04 ETB from both permanent and temporary migrants. This can be related to the contrasting effects of migration and remittances. Overall, total household income, the share of crop income, remittances, landholding, and livestock value for migrant households vary across temporary and permanent migration alternatives Table 2.

**Table 2:** Household income shares and asset holdings, by migration status.

Variable mean	Total samples (n = 795)	No migration (n = 513)	Temporary migration (n = 123)	Permanent migration (n = 150)
Average land holding size (ha)	1.72	1.61	1.95	1.89
Average land devoted to crop cultivation (ha)	1.05	0.97	1.32	1.08
Share of crop income (%)	34.15	58.25	22.90	15.31
Share of self-employed income (%)	2.57	3.66	3.02	2.19
Share of wage/other incomes (%)	1.23	2.49	1.16	0.69
Share of remittances (%)	23.39	---	59.16	71.85
Households living below the poverty line (%)	27.80	40.94	6.92	1.32
Value of livestock ownership (ETB)	11806.30	12814.98	9563.30	10320.25
Rural household income, total (ETB)	56743.20	35628.84	86443.13	102602.90

Source: Authors’ survey.

Note: Means in this table are estimated at the individual level.

Though complete labor substitutes are not likely to be available in underdeveloped countries the decision to send out migrants can constrain high-return activities. This may result in a substantial adverse effect on the rural economy [8, 9, 15, 16, 38]. We find that incomes from specific sources are typically lower for households with migrants than households with no migrants. On average, migrant households have earned about 4,416 ETB (21.1%) less crop income than the average households with no migration. In addition, the value of livestock holding is lower for migrant-sending households. Given the competing differences between migrant and non-migrant households for some variables, other variables may confound the descriptive analyses. We applied empirical methods explained in the next section to examine the nexus between migration, remittances, incomes, and asset ownership in migrant source households while controlling the potential effect of confounding variables.

## Econometric methods and data

### Econometric model

Under close consideration of NELM theory, we adopted an empirical model developed by Stark [39] then applied by Rozelle, Taylor [9]; Taylor, Rozelle [38]; Quinn [15]; and Li, Wang [8]. The NELM theory predicts that migration decisions are made at the household level in which family members of the household act collectively to maximize income and minimize risks and loosen constraints associated with market failures. Specifically, Stark [39] hypothesizes that migrant remittances play the role of financial intermediaries, enabling rural households to overcome credit and risk constraints on their ability to achieve the transition from familial to commercial production. By applying these insights, we develop a model aimed to evaluate how migration and remittances affect incomes and asset ownership through overcoming production constraints faced by households in poor country environments.

We assumed that if the production of high-return activities is constrained and migration, M, and remittances, R, affecting production constraints, then the constrained vector of household income streams, Y^*c*^, depends on M and R, in addition to the individual, household, and community level variables, X_*i*_. Through production, migration and remittances may have various effects on different household income sources. We define household income sources other than remittances, as crop income, Y_*c*_; self-employed income, Y_*s*_; and wages and other local incomes, Y_*w*_. Rural household income is the sum of the remittances and other sources of income as specified above. The main equation of our empirical model is specified as follows:

$$Y_{i}^{c}=\alpha_{0i}+\alpha_{1i}M_{t}+\alpha_{2i}M_{p}+(\alpha_{3i}+\alpha_{4i}M_{t}+\alpha_{5i}M_{p})R+\alpha_{6i}X_{i}+\varepsilon_{i};\;i=c,\;s,\;w$$ (1)

Similarity, we also estimate the impact of migration and remittances on asset accumulation, A_*a*_: defined in terms of land owned, include the net rented land, A_*l*_; and the value of total livestock holdings, A_*v*_; and modeled as:

$$A_{a}=\beta_{0a}+\beta_{1a}M_{t}+\beta_{2a}M_{p}+(\beta_{3a}+\beta_{4a}M_{t}+\beta_{5a}M_{p})R+\beta_{6a}X_{a}+\varepsilon_{a};\;a=l,\;v$$ (2)

As we want to control the impact of migration depending on the type of migration, we first specify the migration decision, M, as a binary endogenous variable which assumes the value of one if the n^*th*^ household participates in migration and zero otherwise. We further mapped migration into two different categories and each migration categories has a value of one if the household participates in one of the migration alternatives: temporary migration, M_*t*_, or permanent migration, M_*p*_, and zero if no migration. We also estimate the effect of remittances R (the sum of remittances sent by both temporary and permanent migrants) on outcome variables. To further account separate effects, we add the interaction of the remittances with the migration typologies: (a) remittances and temporary migration, and (b) remittances and permanent migration. We believe that the migration and remittance trends in these sample areas reflect the national migration stock and capture the inherent economic and demographic characteristics of the households.

Importantly, we assumed that the potential impact of migration is complex. The decision to send out a migrant can lead to the loss of human and financial capital. Thus, keeping all explanatory variables constant, neither migration nor remittances affect household income streams (H_0_: α_1_ = 0, α_2_ = 0, α_3_ = 0, α_4_ = 0, and α_5_ = 0 ∀ *i*, against H_1_: H_0_ is not true). The same holds for the equation of asset accumulation (2). It’s unlikely that all households sending out migrants receive remittances or not, all the received remittances are invested in high-return activities.

Further, to model the determinants of migration decision and participation in different migration alternatives, different statistical techniques are required depending on the type of the outcome variable. The first measure is a binary outcome variable; whether any member of the household migrates at least for three months preceding the survey period, or not. Logistic regression with odds ratio is the proper method for this dependent variable. The second set of migration measure is the probability of a household to have a migrant in one of the two migration categories that is temporary and permanent migration with respect to the option of not migrating. We adopted a multinomial logistic model as the dependent variable has more than two nominal categories. Assume migration, M, is the binary outcome variable which takes the value of one if the household had at least one migrant member and X be a set of factors influencing the decision to migrate, including temporary migration, M_t_, and permanent migration, M_p_, whereas Z_M_ stands for instrument variables:

$$M=\delta_{0}+\delta_{1}X_{M}+\delta_{2}Z_{M}+\varepsilon_{M}$$ (3)

$$M_{t}=\theta_{0}+\theta_{1t}X_{M}+\theta_{2t}Z_{M}+\varepsilon_{M}$$ (4)

$$M_{p}=\mu_{0}+\mu_{1p}X_{M}+\mu_{2p}Z_{M}+\varepsilon_{M}$$ (5)

Besides, the sum of remittances R is produced by allocating family members to labor migration, M, including both migration choices and other human and household factors that motivate the migrant to remit some amount of their income share back to origin home, X_R_.

$$R=\gamma_{0}+\gamma_{1}M_{t}+\gamma_{2}M_{p}+\gamma_{3}W_{R}+\varepsilon_{R}$$ (6)

Where W_R_ stands for community-level instrument variables and Eq (6) is estimated using OLS with robust standard errors.

### Estimation methods and issues

Nevertheless, several studies have explicitly examined the relationship between migration and incomes and asset accumulation in source communities [6, 12, 38], our study allows the impact of migration and remittances to be different for households with different migration choices and test whether the reduced labor availability and the consequent remittances are more relevant for permanent than for temporary migrants. In addition, numerous studies have examined the factors that determine a household’s migration decision and its socio-economic impact independently and vice versa. The most-common economic model for migration [40, 41] has no place for the effects of remittances in migrant source communities.

Our empirical analysis is twofold and aims to understand the factors that determine the household’s migration decision and the various impacts migration have on household income and asset accumulation in origin areas of migration. To achieve these objectives, we carried out two phases of statistical analyses. First, using logistic regression we estimate the determinants of the household’s migration decision of having migrants. Since there are different types of migration which yield different levels of net remittances, using multinomial logistic regression we estimate the household’s behavior towards the probability of having migrants in either temporary or permanent migration alternatives. Second, to empirically test the impacts of migration (temporary and permanent migration) and the subsequent influx of remittances on income sources and asset ownership of smallholder farm households we adopted three-stage least-squares method accompanied by endogeneity and multicollinearity test to account the potential simultaneous correlation issues stemming from both observed and unobserved characteristics.

The methodological challenges in estimating the impact of migration and remittances using observational studies are, however, to construct a counterfactual situation against which the impact can be measured because of selection bias related to migration decisions and remittance recipients [42]. The migration decision is observed for all households, while remittance receivers are only observed for households that have migrants. In the current study, however, selectivity bias is not a serious issue because 35.47% of the randomly interviewed households had at least one migrant and off these migrant households, 86.2% had received remittances. Further complicating the estimation is migration and remittances are expected to be both endogenous with respect to the household’s production decision on high-return activities. Finally, migration, remittances, and household income sources may be subject to a reverse causality which could lead to simultaneous correlation across equations.

Due to the limitations in our dataset, we are able to identify the system and control many of the issues that arise from endogeneity and selectivity bias across Eqs (1) to (6) using instrumental variables. A good instrumental variable, one that is correlated with the suspected explanatory variable but uncorrelated with the outcome variable, can eliminate many of the biases that arise from endogeneity, selection bias, and omitted variables. More recently, Bettin et al. [43] have proposed an instrument variable test in the empirical literature, where the potential selection bias of migration and remittances is taken into account. There are two vectors of instruments in this study, Z_M_ for the migration Eq (3) and W_R_ for the remittance Eq (6). We have identified instrument variables that explain the dependent variable they are instrumenting (migration and remittances) but uncorrelated with the outcome variable (household income sources and asset ownership).

Migration networks and migration experiences are frequently used in the literature to instrument migration see for example [15, 16, 38]. The presence of a national community at the destination could drive migration by reducing the monetary and non-monetary cost of migration [44]. Further, it is more likely that rural out-migration increases with the prevalence of high share of adults with migration experiences in source communities [15]. We adopted these variables to our analyses: first we create an indicator which assumes the value of one if a household in the village sends out a migrant in 1991, as proxy measure for migration networks and second, we identify the proportion of adults from the community with migration experience, as a proxy measure for the previous migration experience of the sample villages. We hypothesize that migration networks and the percentage of adults with migration experience at the village level affect the stock of migrants at the household level, but do not have direct impacts on outcome variables.

Remittances are produced by allocating some family members to migration. In addition, household characteristics and community variables affect the trend of remittances inflow. We use village-level variables, such as migrants return (if households in the village experienced migrants return for Ethiopian New Year) and migrant contribution to a church service (whether the migrant contributes to funding church services in the home community) as instrumental variables to predict the amounts of remittances received by the household. If migrants return home or intend to visit their families in the village during public holidays, they may bring some share of their income to invest in land, livestock, and they may also bring some gifts in kind to build their reputation in origin communities. Traditionally, Ethiopian migrants also contribute to funding community churches and a considerable amount of remittances is often sent back to origin communities to fund church service. We assume these village-level factors affect each household’s remittance level but have no independent effect on household decisions regarding the production of high-return activities.

Finally, the stochastic error terms ε_*i*_, *i* = *c*, *s*, *w*, R, M are assumed to be normally and independently distributed with mean zero and variance $\sigma_{i}^{2}$. In fact, correlation across-equations is more likely to occur, as many of the decisions on migration, remittances, and investment decisions on high-return activities are made at the same time as other household decisions. To this end, we apply an iterated three-stage least-squares (3SLS) method to regulate the potential instantaneous covariance issues across equations.

### Data and variables

**Fig 2:**
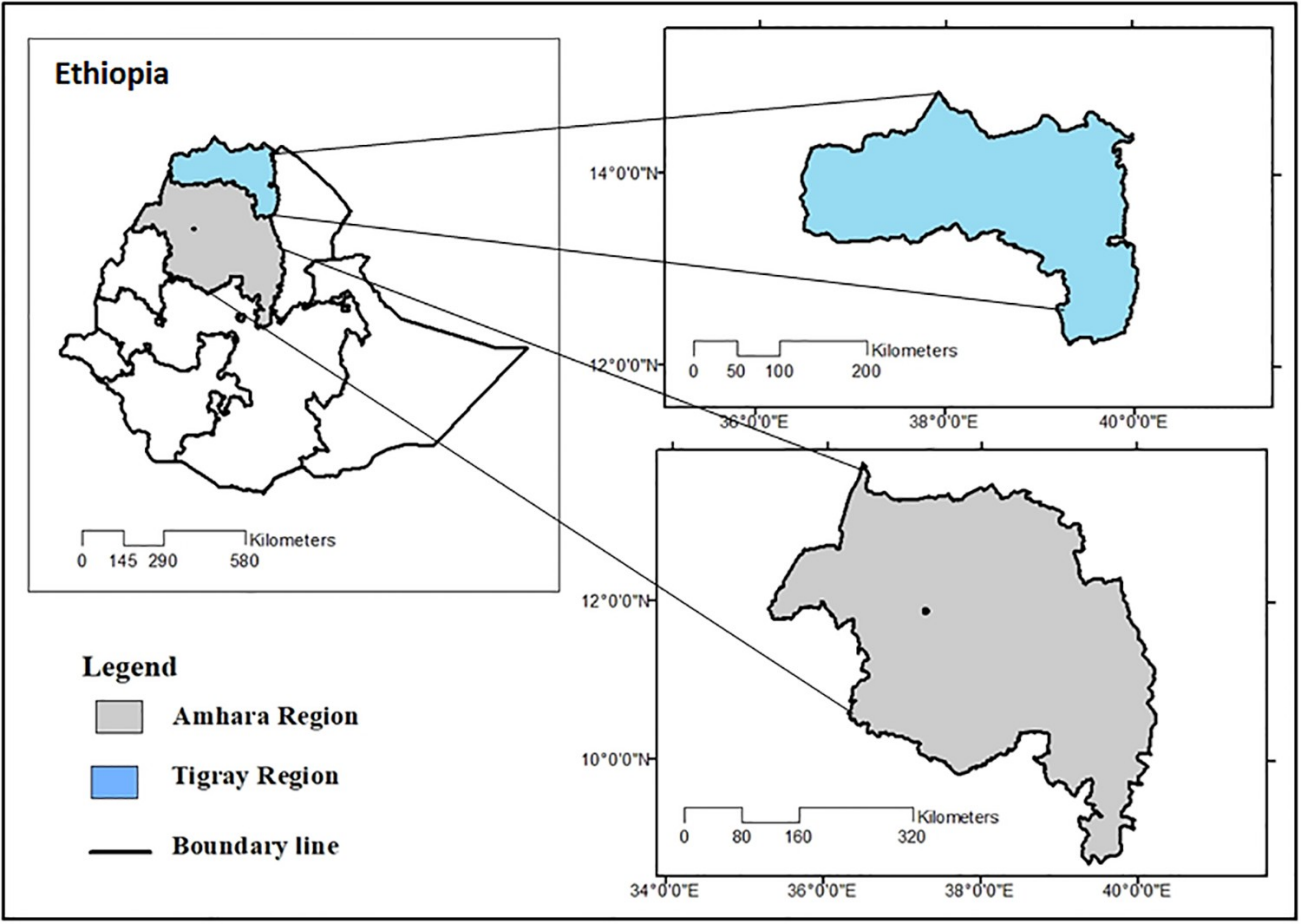
Map of the study areas. Source: Author’s own survey.

The empirical analysis is based on a survey of 795 farm households from seven villages of northern Ethiopia. The data were collected during the summer of 2016 with the purpose of analyzing the developmental impacts of migration and remittances in migrant-sending households. To represent the agriculture and migration trends of the study area different weights are allotted to each region, zone, district, and village under the consideration of various combinations of topographic and economic features. The sampling design includes seven major agriculture villages distributed in two major administrative regional states, Tigray and Amhara (Fig 2). A multi-stage stratified sampling procedure was used to select sample villages from each survey area and households from each village. The survey collected detailed information on household composition, human capital, physical assets, incomes, migration, and remittances.

Migrants were identified from the household survey as family members who have left the household to work elsewhere for at least three months prior to the survey period. The occurrence of a large number of migrants in the study areas which tally to 16.8% of the national migration stock enables us to find a tolerable number of migrants in the sample households. Of the total sample households, 35.47% (282 observations) had sent at least one family member for migration, 15.47% (123 observations) have participated in temporary migration and 18.87% (150 observations) had sent at least one family member for permanent migration. Only 1.11% (nine households) had both temporary and permanent migrants. Of these migrant-sending households, 86.2% (273 observations) had received remittances.

Since the total number of migrants and the amount of remittances does not affect outcome variables, in the same way, migrant households identified through a household questionnaire are then mapped into two sub-samples; households with temporary migrants: a moving for short periods of time, usually for less than a year and do not change primary residence; and households with permanent migrants: a moving for long periods of time, usually for 12 months or more and do change primary residence [45]. We believe that the migration and remittance trends in these sample areas reflect the national migration stock and capture the inherent economic and demographic characteristics of the households.

The dependent variables of interest are defined as follows. Household incomes other than remittances are attributed to three possible income streams, such as crop income includes all earnings from crop sales; self-employment income includes all proceeds from conducting local business operations; and wages and other incomes: any local off-farm wages and other incomes earned by the household. The sum of remittances and all incomes equal household income. Asset accumulation is defined in the form of farm size (at least 0.1 hectares) and current values of livestock ownership. The survey also covered a wide range of controlled variables that influence the choices of high-return farm activities at household level Table 3.

**Table 3:** Description of outcome, explanatory, and instrument variables (N = 795).

Variables	Definition of variables (units)	Mean	Std. Dev.
Outcome variables			
Crop income	The sum of proceeds from the sale of crops, fewer expenses (ETB)	19374.91	12798.78
Self-employed income	The proceeds from conducting local business operations (ETB)	1461.38	2283.07
Wage and other income	Local wages and other incomes earned by the household (ETB)	701.69	1167.27
Value of livestock	The value of all livestock units owned by the household (ETB)	11806.30	9772.71
Landholding size	Cultivated farmland owned by the household (ha)	1.72	0.98
Both dependent and independent variables			
Migration	If a household sent at least one migrant to work elsewhere (yes = 1)	0.35	0.48
Temporary migration	If the household had at least one temporary migrant (yes = 1)	0.16	0.37
Permanent migration	If the household had at least one permanent migrant (yes = 1)	0.19	0.39
Remittances	If a migrant-sending household received remittances	0.86	0.34
Log value, remittances	Log value of remittances received by migrant households in ETB	1.39	1.99
Explanatory Variables in X_i_			
Age of the HH head	Age of the household head in completed years	50.28	12.32
Gender of the HH head	Dummy indicating the gender of the household head (male = 1)	0.72	0.45
Family size	The number of current family members in the household	6.21	2.31
The share of working age	The proportion of working age in the household	40.67	19.38
Young dependents	Number of children under the age of 18 years	1.98	0.85
Level of education	The education level of the household head in schooling years	5.98	3.41
Value of assets	Log value, non-productive household assets	2.15	3.78
Household living below the poverty line	If the household is living below the international extreme poverty line of US$1.90 a day, as of 2016	0.28	0.55
Unemployment	Average percentage of unemployment rate at village level	10.08	6.15
Access to irrigation	If the household is accessed to irrigation schemes (yes = 1)	0.53	0.50
Nearest to the main road	Near to the road where a farmer obtains transport service (yes = 1)	0.82	0.38
Instrumental variables for migration			
Migration networks	If a household in the village sends out a migrant in 1991 (yes = 1)	0.29	0.46
Migration experience	The proportion of adults in the community with migration experience	10.67	17.04
Instrumental variables for remittances			
Migrants return	If households in the village experienced migrants return (yes = 1)	0.29	0.45
Migrants church	If migrants contribute to fund church in home community (yes = 1)	0.32	0.47

Source: Authors’ survey.

## Ethics statement

In the data collection process, the household survey was conducted anonymously. Research and Ethical clearances were obtained from Nanjing Agricultural University; Office of the International Education (April 27, 2016) and the Ethiopian Ministry of Education, Office of Foreign Affairs and Scholarship Directorate (Ref: 12-/2-4942/1439/35). Permissions were also granted from Local and Regional Authorities in Ethiopia. All research participants were adult head of the sample households. With the help of local development agents, surveying procedures were explained to each participant and verbal consent was obtained from all participants. Due to the limitations of available resources, we were only able to get verbal consent and it was documented using a tape recorder for every participant. Independent community members, called community constabularies of every survey village have approved the consent and acted as a witness for voluntarily informed decision making of participants to take part in the study. Ethics committees were aware that minors (under 18 years) would provide their own consent. Overall the data collection procedures and indeed the data analysis and depository were made anonymously in a way that prevents survey participants from being identified by name and number.

## Empirical results and discussion

### Estimating the determinants of migration choice

Table 4 shows the estimated effects of household and village characteristics on household decision to have migrant family members, Eq (3), and whether the household has participated in different migration alternatives, Eqs (4) and (5). Two alternative specifications were used, first with each of the instrumental variables we estimate a logistic regression, Eq (3) to identify the probability of a household to have a migrant (overall migration decision) in col.1, and second, we estimate the multinomial logistic regression predicting the probability of a household to participate in one of the two types of migration alternatives with respect to the option of not migrating, Eqs (4) and (5) in col. 2 and 3. The predictions from the migration equations are the first stage results and will be used in an instrumental variable test explaining income sources, land ownership and the value of livestock. All specifications yield parameter estimates that are largely consistent with the expected effects.

**Table 4:** Estimating the effects of household and village characteristics on household’s decision to have migrants in different migration alternatives (n = 795).

Explanatory variables	Migration decision		Temporary migration	Permanent migration
	(1)	Odds ratio	(2)	(3)
Household characteristics				
Gender of the household head	-0.343	0.709	-0.098	0.127
	(0.294)		(0.267)	(0.278)
Current family size	0.089	1.093	0.119	0.061
	(0.173)		(0.190)	(0.276)
Percentage of working age	0.133	1.142	0.208	0.167**
	(0.165)		(0.187)	(0.273)
Young dependents (<18 years old)	0.837***	2.256	0.201	0.112
	(0.283)		(0.340)	(0.368)
Education level of the household head	0.092**	1.097	0.089**	0.021
	(0.041)		(0.035)	(0.036)
Land holding size (hectares)	-0.355**	1.257	0.202	-0.094**
	(0.149)		(0.131)	(0.141)
Value of livestock holdings	-0.063***	0.393	-0.026*	-0.008
	(0.015)		(0.016)	(0.013)
Log value, household assets	0.239***	1.271	0.020	0.081***
	(0.033)		(0.031)	(0.031)
Village characteristics				
Household living below the poverty line	0.579***	2.375	0.479	0.780***
	(0.502)		(0.415)	(0.644)
Percentage of the unemployment rate	0.357***	1.285	0.109***	0.055**
	(0.026)		(0.028)	(0.027)
Migration network, out-migration occurring in 1991	0.371***	1.231	0.442***	0.252***
	(0.054)		(0.491)	(0.422)
Percentage of adults in the village with migration experience	0.103***	0.059	0.152**	0.171***
	(0.023)		(0.096)	(0.171)

Source: Authors’ survey.

Note: Coefficients are presented with robust standard errors in parentheses. Whereas ***, **, and * denote the significance level at 1%, 5%, and 10%, respectively.

For example, wealthier households (measured as larger owners of farmland and livestock units) are less likely to have migrant family members in either type of migration. Whereas households living below the poverty line, as well as households with the highest unemployment rate, are more likely to participate in both temporary and permanent migration alternatives. The percentage of working age has a positive effect on the probability of having a permanent migrant in the past year. Households having larger young dependent members were also more likely to have a migrant in the past year. Most educated households are about 9.7% more likely to have a migrant and particularly engaged in temporary migration. The actual value of household assets is quite positively correlated with high potential for permanent migration. Besides, village characteristics, such as migration networks and migration experience are found to have significant positive effects on migration choices. Households in villages with large migration networks and migration experience are more likely to have migrants in both temporary and permanent migration alternatives.

Our results provide support for the NELM theory that larger asset owners and wealthier households found retaining the potential migrants locally would be more worthy, infers that the perceived returns to migration would be intended to support in overcoming production constraints. By contrast, households living below the poverty line and villages with the highest unemployment rate are more likely to have both types of migrants, indicating that the initial loss of labor effects and other costs associated with migration are not an issue for poor households since migration has long been an essential option to maximize household income for the poor rural farmers. Despite poor households face migration constraints and are often unable to migrate, we observed that extreme poverty is a fundamental driver of rural out-migration. Our results are consistent with the findings of other scholar see [17, 21, 46].

### Multicollinearity test

To further account multicollinearity issues among migration and remittance variables and in fact, to control the linear correlation issues between the instrument variables we have applied the procedures of multicollinearity test. The primary concern is that as the degree of multicollinearity increases, the regression model estimates of coefficients become unstable and the standard errors for the coefficients can get widely inflated. In the sample (and therefore in the population), none of the independent variables is constant and there are no exact relationships among the independent variables. The assumption only rules out the perfect relationship between explanatory variables. The most useful way to detect the problem of multicollinearity in a given application is the variance inflation factor (VIF). The VIF can tell us the extent to which the standard errors of the coefficient of interest have been inflated upward. As a rule of thumb, we do not want that the standard errors to have been inflated more than twice its basic size.

S2 Table shows the results of multicollinearity test for migration and remittance variables. Respectively, the VIF of 9.52 and 6.45 for migration and remittances indicates that there is a multicollineariy problem with in these variables. It is not surprising as remittances are produced by allocating family members to migration. 3SLS method solves this problem by identifying instrument variables that can be used to estimate migration and remittances but uncorrelated with outcome variables. By contrast, the VIF for instrumental and controlled variables looks fine indicates that there is no multicolliearity problem with in the instrumental and controlled variables in the migration and remittance models S2 Table.

### Three-stage least-squares (3SLS)

Many of the decisions about migration, remittances, and investment in high return activities are usually made simultaneously at the family level as part of the livelihood strategy to maximize household income. Household characteristics that cause migration may also shape household patterns of production choices. Given their complex relationship, neither variable can be included instantaneously as an exogenous variable in the main Eqs (1) and (2). In this case, the two suspected variables (migration and remittances) are tested separately for endogeneity. As expected, the results indicate that migration and remittances are found to be endogenous with respect to all specifications of the outcome variables. For instance, the null hypotheses of exogeneity for both migration and remittances with respect to crop income and asset holdings can be rejected at 95% confidence level S1 Table.

To determine these issues, the findings shown in Table 5 are estimated using the iterated 3SLS method. The estimator performs reasonably well and the R^2^ statistics for all the outcome variables are significantly different from zero. The instruments also pass the Hausman-Wu test for endogeneity, using the overall migration decision, as well as the alternative migration specifications. It is interesting to note that the decision to migrate and alternative migration choices make some difference at this point. In general, the results find some evidence to support both the migration and remittances hypotheses, though the coefficients on migration and remittances are found to have mixed results. The exogenous variables defined in the previous section also affect migration, income sources and asset ownership in ways that are consistent with findings by other similar studies. For instance, households with more shares of working age, large land size and access to irrigation are able to generate higher crop income. Households with large values of livestock units are more able to generate self-employment income and expand farm areas. The education level of the household head finds to be positively associated with local wage and other income. In addition, nearest to the local market is quite positively correlated with the higher value of livestock units, whereas female-headed households generate less income from self-employment than their male counterparts do.

**Table 5:** Estimating the effects of migration and remittances on household incomes and asset holdings, using 3SLS (n = 795).

Explanatory variables	Crop income, all crop sales	Self-employed income	Wages and other incomes	Landholding size in ha	Value of livestock units
	(1)	(2)	(3)	(4)	(5)
Temporary migration	332.2	782.2**	29.95	0.753	455.4**
	(250.2)	(441.9)	(23.13)	(0.188)	(190.6)
Permanent migration	-968.4**	108.35	32.22	-0.786**	-382.3***
	(414.6)	(73.22)	(38.33)	(0.314)	(316.7)
Remittances*temporary migration	0.617	0.515	0.445	0.007**	0.021
	(0.779)	(0.674)	(0.493)	(0.003)	(0.029)
Remittances*permanent migration	1.047***	0.076	0.051	0.063**	1.112**
	(0.539)	(0.095)	(0.049)	(0.409)	(0.412)
Remittances, total	1.359***	0.691	0.108	0.064**	1.933**
	(0.391)	(0.519)	(0.983)	(0.028)	(0.041)
Log value of remittances, total	193.0***	90.31	41.37	0.111***	0.475***
	(72.23)	(127.4)	(66.74)	(0.0547)	(0.551)
Age of the HH head	-32.65	0.448	-3.883	-0.002	0.005
	(36.61)	(6.464)	(3.384)	(0.003)	(0.028)
Gender of the HH head	330.4	-310.5*	-122.8	-0.043	-0.722
	(994.8)	(175.7)	(91.96)	(0.076)	(0.760)
Current family size	375.9	-181.4	-53.03	0.012	-0.323
	(580.6)	(102.5)	(53.67)	(0.044)	(0.444)
Percentage of working age	275.5**	135.1	52.98**	-0.041	0.658
	(582.0)	(102.8)	(53.79)	(0.044)	(0.444)
Young dependents	-920.0	337.1	72.99	-0.080	-0.301
	(972.5)	(171.7)	(89.90)	(0.074)	(0.743)
Level of education	95.13	11.99	27.51***	0.002	0.080
	(13.15)	(23.22)	(12.15)	(0.010)	(0.100)
Landholding size	427.0***	-101.8	-10.96		0.171
	(466.8)	(82.42)	(43.15)		(0.357)
Value of livestock	28.66	15.33***	5.198	0.002***	
	(46.40)	(8.193)	(4.289)	(0.003)	
Access to irrigation	417.5*	112.7	24.08	0.021	-0.082
	(900.0)	(158.9)	(83.20)	(0.068)	(0.688)
Nearest market	1390.5	103.7	34.08	0.132	163.9*
	(1185.9)	(209.4)	(109.6)	(0.090)	(0.904)

Source: Authors’ survey.

Note: Coefficients are presented with standard errors in parentheses. Whereas ***, **, and * denote the significance level at 1%, 5%, and 10%, respectively.

### The effects of migration and remittances on income sources and asset accumulation

We estimate the diverse effects migration and remittances have on income sources, as well as on asset accumulations. We find that the direct impact of migration is not likely to be equal for households with different types of migration, as the decision to send out temporary and permanent migrants have sharply different net-returns in terms of initial negative effects and the positive impacts it produces. Remittances are a positive function of migration. Each additional temporary migrant is associated with a 1,034 ETB increase in remittance income, whereas permanent migrants linked with 1,736 ETB rise in remittances S3 Table.

Although the influx of remittances that permanent migrants send back home boost household income and asset ownership, our results indicate that remittances come at the expense of farm labor removal. Specifically, crop income falls significantly, but only for permanent migration. Holding other factors constant, crop income declines by 968.4 ETB for a permanent migrant-sending household. Losing farm laborers to permanent migration also finds to have a significant negative effect on asset accumulation. Permanent migration in the previous period leads to a reduction in farm areas by 0.786 hectares, as well as the value of livestock units fall sharply by 382.3 ETB. The possible explanation is that permanent migration denies valuable labor force for extended periods and threatens the capacity of households to respond to labor demands, leading to a decline in crop income, landholding and livestock ownership. By contrast, incomes from local business and livestock sales increase with temporary migration, though crop income and local wages are invariant. The positive effects do not come as a surprise because temporary migration does not induce a lost-labor effect as it usually implies a kind of circular short-term moves that allow laborers to return home with their earnings to work in origin farm areas Table 5.

While permanent migration has a negative effect on household income sources and asset accumulation, the influx of remittances partially compensates for this. Particularly, each ETB remitted by permanent migrants is associated with 1.047 ETB of additional crop income, 0.063 hectares of additional landholding size and 1.112 ETB of additional livestock value. Land ownership also increases with remittances received from temporary migrants, though all incomes and livestock value remains invariant. The sum of remittances received from both temporary and permanent migrants have also a significant positive impact on crop income and asset ownership. Each ETB remitted by migrants is associated with 1.359 ETB of additional crop income, 0.064 hectares of additional land and 1.933 ETB of additional livestock value.

Besides, Table 5 reports estimates of the average response of incomes and asset ownership to the elasticity change in remittances. We see that the estimated coefficients of log value of remittances on crop income and asset ownership are positive and statistically significant at 99% confidence level, suggesting that remittances support household income and productive asset holdings in rural areas. Quantitatively, a percentage increase in remittances is associated with 193.0 ETB increase in crop income, 0.111 hectares increase in landholding size, and 0.475 ETB increases in livestock value.

Overall, the results suggest that although losing farm laborers to migration reduce high-return farm activities and tighten labor shortage for ranching livestock the remittances sent back by migrants partially relax household’s capital constraints and enables them to engage in high-return production activities compared to households with no migrants. However, based on the above results we can confidently say that at least in the case of crop income, landholding size and value of livestock, migration and remittances have complex effects in migrant source communities.

### Estimating the net effects of migration

Overall, our results support the NELM hypotheses that remittances offset lost-labor effects and loosen production constraints on household income sources and productive asset accumulations. Migration has, however, multiple effects on crop income and productive assets, particularly in the case of permanent out-migration. We estimate the total net effects of permanent migration in migrant-sending households to account for these complexities. The net effect is a sum of direct (labor-loss) and indirect effects (remittances). To estimate the net effects of migration on rural household income and productive household assets, we use a bootstrap method. Using bootstrapping, we produce confidence intervals around the expressions that measure the net effects of migration for households sending out permanent migrants Table 6. Since temporary migration is found to be positively related with outcome variables, we did not conduct a bootstrap for this.

**Table 6:** Bootstrapped net effects of migration on household income sources and asset ownership.

Derivatives/bootstrap	Observed estimates	Bootstrap std. errs.	95% conf. interval
Net effects of permanent migration on crop income and productive assets			
∂Y_c_/∂M_p_	783.1	313.9	[139.8, 1678]
∂A_l_/∂M_p_	0.712	0.288	[0.151, 1.274]
∂A_v_/∂M_p_	251.5	217.5	[67.77, 747.8]
Net effects of migration on total rural household income			
∂Y/∂M	800.8	851.2	[346.9, 1323.8]

Source: Authors’ survey.

Note: In this table, M_p_ stands for permanent migration, and Y_c_, A_l_, and A_v_ stand for crop income, landholding size, and value of livestock, respectively.

Findings suggest that as migrants leave the household permanently, but remit some amount of their income share to origin families, the proportion of crop income increases by a net of 783.1 ETB. Besides, the entire 95% confidence interval is positive. When we consider the total derivative of rural household income with respect to migration, we also find that the total household income increases by a net of 800.8 ETB. Similar to crop income results, we find that an increase in remittances significantly affects productive asset accumulations, land size and the value of livestock increases by a net of 0.712 and 251.5 ETB, respectively. Importantly, migrant remittances compensate for the multiple lost-labor effects of permanent migration. This result is not surprising because due to the high prevalence of imbalance between the supply of and demand for workers in rural Ethiopia, the family member would have the lowest marginal product of labor on local agricultural production. Finally, a positive point estimate would suggest that the total income and asset accumulation for origin household are higher after migration participation.

## Conclusions

This article evaluates the development impacts of migration and remittances in migrant source communities by applying insights from the NELM theory to Ethiopia’s migration. Using household survey data, we empirically evaluate how household participation in migration arises and so that migration impacts income sources and productive assets. The prevalence of high migration stock in the study areas indicates that migration has long been an important livelihood diversification strategy in rural Ethiopia. We find that smallholder rural farmers largely diversify their income streams through migration because the poor economies of scale severely limit their ability to compete in the market and they often do not have sufficient resources to survive risk and income shocks, as about 28% of them are living below the poverty line.

A contributing factor to this view relates to economic and social factors. Findings suggest that compared to smallholders, larger and wealthier households are less likely to have migrant family members in one or the other type of migration, while households living below the poverty line, as well as villages with the highest unemployment rate typically involved in temporary and permanent migration alternatives. Households with the highest percentage of working age and young dependents are also featured as the most migratory one. Further, educated households are about 9.7% more likely to have a temporary migrant. The actual value of household assets is quite positively correlated with the potential to have a permanent migration as well.

On the other hand, we provide evidence that the direct impact of rural out-migration at the household level varies for households with different types of migration. For instance, the loss of farm laborers to permanent migration leads to a significant negative effect on crop income, land size, and value of livestock units. Specifically, crop income, landholding, and the value of livestock units fall sharply by 968.4 ETB, 0.786 hectares, and 382.3 ETB, respectively. By contrast, the departure of family members for temporary migration has a significant positive effect on self-employed income, land size, and value of livestock units, but has no effect on crop and local wage incomes. These findings suggest that the heterogeneous effects of migration are only relevant for households with permanent migrants.

While permanent migration has a negative effect on crop income, land size and livestock values, the influx of remittances can partially compensate for the negative impacts induced by the reduction of family labor availability. Particularly, each ETB remitted by permanent migrants is associated with 1.047 ETB of additional crop income, 0.063 hectares of additional landholding size and 1.112 ETB of additional livestock value. Furthermore, the total remittances received from both migrants have a significant positive impact on crop income and asset accumulation in terms of landholding size and value of livestock ownership. Quantitatively, a percentage increase in remittances is associated with 193.0 ETB increase in crop income, 0.111 hectares increase in landholding size, and 0.475 ETB increases in the value of livestock. The net impact of migration would suggest that household income and asset accumulation for a source household, are higher after migration participation.

We conclude that migrant remittances combined with local production conditions have a notable impact on smallholder agriculture outcomes in remittance-receiving households. The influx of remitted income has led to increased crop income and land acquisition. In addition, smallholder farmers appear to utilize economic migration as a means to accumulate assets in the form of livestock. Under such circumstances, investments in high-return activities and asset accumulation consistent with a decreasing available family labor make economic sense.

Finally, although migration from developing countries has become a prominent issue of economic development, the policy issues facing today’s national leaders is whether this process should be promoted or discouraged. The massive migration of labor out of agriculture leads to a reduction in smallholder agricultural production including crop and livestock production, potentially threatening Ethiopia’s food security. If the Ethiopian government wishes to slow the massive flow of migrants out of agriculture, it may call for effective policy interventions by reforming the formal and informal rural micro-finance institutions. Such measures would increase households’ self-employed production efficiency and reduce the desire to send out migrants into the labor market primarily to finance these activities. However, more thorough reforms to institutions, such as lifting the restrictions in private sector, will likely to retain the migration of massive laborers out of agriculture and ensure that youth remain socially and economically engaged and productive in the local agricultural economy.

## Supporting information

S1 Table Durbin-Wu-Hausman test results for different specifications. (DOCX)

S2 Table Multicollinearity test for migration and remittance variables. (DOCX)

S3 Table Estimating the effects of household and village characteristics on remittances, and interactions between migration and remittances. (DOCX)

S1 File De-identified data. (XLSX)

S2 File Survey questionnaire. (RTF)
