# Supplementary material for: Development impacts of migration and remittances on migrant-sending communities: Evidence from Ethiopia
Source: PLoS One. 2019 Feb 6;14(2):e0210034. doi: 10.1371/journal.pone.0210034 (PMC6364874; doi:10.1371/journal.pone.0210034)
Supplement: S1 Table — (DOCX) [file pone.0210034.s001.docx]

**S1 Table. Durbin-Wu-Hausman test results for different specifications**

| Endogenous variables | Tests | Specifications | | | | |
| --- | --- | --- | --- | --- | --- | --- |
|  |  | I | II | III | IV | V |
| Total number of migrants, migration | F(1,782) | 2.274 | 2.064 | 0.132 | 3.824 | 4.287 |
|  | p-value | 0.034 | 0.059 | 0.716 | 0.051 | 0.023 |
| Total amount of remittances | F(1,782) | 2.427 | 0.102 | 0.326 | 10.351 | 2.199 |
|  | p-value | 0.091 | 0.749 | 0.568 | 0.001 | 0.039 |
| Households with temporary migrant | F(1,782) | 11.44 | 27.00 | 0.933 | 4.813 | 5.384 |
|  | p-value | 0.001 | 0.000 | 0.334 | 0.028 | 0.021 |
| Households with permanent migrant | F(1,782) | 4.369 | 16.08 | 1.887 | 5.483 | 8.483 |
|  | p-value | 0.037 | 0.000 | 0.069 | 0.019 | 0.004 |
| Remittances*temporary migrant | F(1,782) | 14.01 | 18.20 | 0.268 | 10.63 | 6.101 |
|  | p-value | 0.000 | 0.000 | 0.605 | 0.001 | 0.014 |
| Remittances*permanent migrant | F(1,782) | 10.19 | 9.899 | 0.602 | 10.46 | 8.687 |
|  | p-value | 0.001 | 0.002 | 0.438 | 0.001 | 0.003 |

Source: Authors’ survey.

Note: Instrumental variables for migration and remittances are tested in 2SLS regression methods independently and the Wu-Hausman estimation results for different specifications are reported; where the specifications are denoted as, I = crop income, II = self-employed income, III = local wages and other income, IV = landholding size, V = value of livestock. We apply a direct test of endogeneity assumption for the suspected variables. According to Davidson and MacKinnon (1993), the Hausman-Wu test for endogeneity is a three-step process. The first step is identifying variables which are supposed to be endogenous—in this case, the two suspected variables are migration and remittances. Next, we run a regression with the suspected endogenous variables as a dependent variable. Then the predicted residuals from this regression are saved and used as an independent variable in the original equation. It is still a good idea to conduct endogeneity test from the 2SLS estimators by a two-step procedure: first, we conduct an IV second-stage least squares (2SLS) regression for all systems of structural equations against all of the exogenous variables, including all of the instruments for the suspected endogenous variables. The primary purpose is to test the presence of endogeneity by comparing the ordinary least squares (OLS) estimate of the structural parameters in the IV regression to that of the 3SLS. Consequently, to decide whether to use an IV analysis compared to a standard OLS analysis we estimate endogeneity (Durbin-Wu-Hausman test results) from the 2SLS regression at the post estimation report for the suspected endogenous variables. The estimated coefficients from the two-step procedure should exactly the same as The Hausman-Wu test for endogeneity in the three-step process. Besides, the instrumental variables are, of course, tested for validity if they explain migration and remittances, but are exogenous to the main outcome variables.
