# Supplementary material for: Development impacts of migration and remittances on migrant-sending communities: Evidence from Ethiopia
Source: PLoS One. 2019 Feb 6;14(2):e0210034. doi: 10.1371/journal.pone.0210034 (PMC6364874; doi:10.1371/journal.pone.0210034)
Supplement: S2 Table — (DOCX) [file pone.0210034.s002.docx]

**S2 Table. Multi-collinearity test for migration and remittance variables**

| **Variables** | **VIF** | **1/VIF** |
| --- | --- | --- |
| Migration decision | 9.52 | 0.105 |
| Remittances, total | 6.45 | 0.155 |
| Migrants contribute to funding church service | 3.68 | 0.272 |
| Percentage of working age | 3.59 | 0.279 |
| Current family size | 3.39 | 0.295 |
| Percentage of adults in the village with migration experience | 3.01 | 0.332 |
| Young dependents (<18 years old) | 2.51 | 0.398 |
| Migration network, outmigration occurring in 1991 | 2.46 | 0.407 |
| Percentage of unemployment rate | 2.38 | 0.420 |
| Households experienced migrants return | 2.12 | 0.471 |
| Households below the poverty line | 1.77 | 0.564 |
| Log value of household assets | 1.24 | 0.808 |
| Value of livestock holdings | 1.20 | 0.831 |
| Landholding size (hectares) | 1.08 | 0.924 |
| Age of the household head | 1.04 | 0.965 |
| Education level of the household head | 1.02 | 0.978 |
| Gender of the household head | 1.02 | 0.983 |
| Mean VIF | 2.79 | |

Source: Authors’ survey.
