# Supplementary material for: Development impacts of migration and remittances on migrant-sending communities: Evidence from Ethiopia
Source: PLoS One. 2019 Feb 6;14(2):e0210034. doi: 10.1371/journal.pone.0210034 (PMC6364874; doi:10.1371/journal.pone.0210034)
Supplement: S3 Table — (DOCX) [file pone.0210034.s003.docx]

**S3 Table. Estimating the effects of household and village characteristics on remittances, and interactions between migration and remittances**

| Explanatory variables | Remittances, total | Temporary migrants  (remittances) | Permanent migrants  (remittances) |
| --- | --- | --- | --- |
|  | (1) | (2) | (3) |
| Temporary migration | 1278*** | 1034*** |  |
|  | (1035) | (1263) |  |
| Permanent migration | 1692*** |  | 1736*** |
|  | (1298) |  | (1549) |
| Age of the household head | 0.005 | 54.05** | 0.019 |
|  | (0.029) | (26.54) | (0.023) |
| Gender of the household head | 0.006 | 0.046 | 0.463 |
|  | (0.866) | (0.898) | (0.666) |
| Current family size | 0.292 | 0.105 | 0.157 |
|  | (0.404) | (0.523) | (0.313) |
| Percentage of working age | -0.146 | 0.067 | 0.087 |
|  | (0.411) | (0.499) | (0.323) |
| Young dependents (<18 years old) | -0.240 | -0.561 | -0.134 |
|  | (0.816) | (0.808) | (0.636) |
| Education level of the household head | 0.096 | -0.030 | 0.082 |
|  | (0.123) | (0.118) | (0.091) |
| Land holding size | 112.8*** | 133.8*** | 624.9** |
|  | (13.38) | (31.79) | (307.9) |
| Value of livestock holdings | -0.013 | 0.032 | -0.008 |
|  | (0.036) | (0.036) | (0.028) |
| Number of summer crop shocks | 400.4*** | 0.067 | 191.4* |
|  | (140.7) | (0.145) | (107.5) |
| Household below the poverty line | 1527* | 0.649 | 1201.6* |
|  | (137.1) | (1.106) | (657.0) |
| Percentage of the unemployment rate | -0.014 | -0.577 | -0.031 |
|  | (0.087) | (0.127) | (0.055) |
| Households experienced migrants return | 65.19*** | 12.86*** | -0.734 |
|  | (19.59) | (2.436) | (0.574) |
| Migrants contribute to funding church | 24.34* | 19.69 | 23.98*** |
|  | (3.499) | (4.212) | (18.25) |
| N | 795 | 795 | 795 |
| R^2^ | 0.906 | 0.793 | 0.925 |
| adj. R^2^ | 0.904 | 0.790 | 0.924 |
| F | 306.3 | 87.01 | 230.8 |

Source: Authors’ survey.

Note: Coefficients are presented with standard errors in parentheses. Whereas ***, **, and * denote the significance level at 1%, 5%, and 10%, respectively.
