# Supplementary material for: Development impacts of migration and remittances on migrant-sending communities: Evidence from Ethiopia
Source: PLoS One. 2019 Feb 6;14(2):e0210034. doi: 10.1371/journal.pone.0210034 (PMC6364874; doi:10.1371/journal.pone.0210034)
Supplement: S2 File — (RTF) [file pone.0210034.s005.rtf]

 
Household Survey Questionnaire    
My name is Misgina Asmelash Redehegn. I am a PhD student in the College of Economics and Management, Nanjing Agricultural University; Nanjing, China. Right now I am doing a research entitled “The Impact of Migration and Remittances on Agricultural Technology Adoption, Household Income and Asset Accumulation in Ethiopia”. This questionnaire is thus developed to gather information required for the accomplishment of this research project. The responses you give are valuable and will be used only for the analysis of this research work. You will not be identified by name in any case. This questionnaire will take about an hour of your time to respond to the questions so you are kindly requested to give your response for the questions listed hereunder.

Thank you in advance for your cooperation!


Required Information
Identification Page	3
Section 1: Household Roster	4
Section 2: Education	5
Section 3: Migration and Remittances	6
Section 4B: Agriculture; Crop Production	9
Section 4C: Agriculture; Harvesting	13
Section 4D: Agriculture; Livestock	14
Section 5: Access to Basic Services	15
Section 6: Annual Income and Household Assets	15
Section 7: Natural Resource Conservation	17

Identification Page
No	1.	2.	3.	4.	5.	6.	7.	8.	9.	
Area 	Region 	Zone 	District	Kebelle/FA	Village	Rural town	Household ID	Sample survey code  	Household name 	
Code 	(_________)	(_________)	(_________)	(_________)	(_________)	(_________)	(_________)	(_________)	(_________)	
10.	
Family Size	
<14 years old	15-19 years old	10-24 years old	25-39 years old	40-64 years old	>64years old	Total family size	
Male 	Female 	Male 	Female 	Male 	Female 	Male 	Female 	Male 	Female 	Male 	Female 	Male 	Female 	Total	
															

NB: Code for study areas will be attached during the survey process! 
Region:                      Zone:           District:            Kebele:          Village:            Town: 
Tigray...............1      
Afar.................2 
Amhara...............3 
Oromia...............4 
Somalie..............5 
Benshagul Gumuz......6 
Snnp.................7 
Gambella.............8 
Harari...............9 
Addis Ababa.........10 
Diredawa............11 
Outside of Ethiopia (specify)...........12

First Interview
11. Date of First Interview:                   /            /                   [Date / Month / Year]
12. Sections incomplete after 1st interview	Cover	Sec-1	Sec-2	Sec-3	Sec-4	Sec-5A	Sec-5B	Sec-5C	Sec-5D	Sec-5E	Sec-6	Sec-7	Sec-8	Sec-7	Sec-8	Sec-9	
																	
																	
Section 1: Household Roster
	1.			2.	3.	 4. 		5.	6.	7.		
I		                    NAME	What is [NAME]'s	What is the sex	 How old is [NAME]?	What is [NAME]'s	 What is [NAME]'s	 Have any of your family		
				relationship to the head	 of [NAME]?			marital status?	 main occupation?	 members ever been 		
												
N												
				of the household?		 IF RESPONDENT DOESN'T KNOW, USE		 	 elsewhere for work at 		
											
D				 1 = Head		 YEAR OF BIRTH TO CALCULATE AGE 	  1 = Never married	 1 = Agriculture
 2 = Mining 
 3 = Professional
 4 = Petty trade
 5 = Construction
 6 = Daily labor
 7 = Other,         
        specify………


	 least for three months? 		
I						 OR USE RECOGNIZED EVENTS			 


 1 = Yes >>SECTION 3
 2 = No

  


		
				 2 = Spouse			  2 = Married 				
		LIST HOUSEHOLD HEAD ON 									
				 3 = Son/Daughter							
V		LINE 1 THEN:					          (mono) 				
				 4 = Grandchild			  3 = Married (poly)				
											
I		  MAKE A COMPLETE LIST OF ALL		 5 = Father/Mother			  4 = Separated 				
							  5 = Widowed				
													
D		 THE INDIVIDUALS AND MEMBERS		 6 = Servant					  6 = Divorced 				
													
		  OF THIS HOUSEHOLD, INCLUDING				 (If 5 years and over, give YEARS only. If					
				 7 = Other, specify………							
U											
		THOSE OF IN LABOR MIGRATION.				 less than 5 years in age give YEARS and					
A											
					1 = Male
2 = Female


	 MONTHS. If less than one month put					
											
L						                         "0"					
											
I		(CONFIRM THAT HOUSEHOLD											
													
		HEAD HERE IS SAME AS											
D													
		HOUSEHOLD HEAD LISTED ON											
		IDENTIFICATION PAGE.)											
													
													
						         Years		Months					
													
1													
													
2													
													
3													
													
4													
													
5													
													
6													
													
7													
													
8													
9													
10													
													


Section 2: Education	


I

N D I V I

D U A L

I

D


1

2

3

4


5

6

NAME


LIST HOUSEHOLD HEAD ON LINE 1 THEN MAKE A COMPLETE LIST OF ALL THE INDIVIDUALS AND MEMBERS OF THIS HOUSEHOLD INCLUDING THOSE OF IN LABOR MIGRATION. 

(CONFIRM THAT HOUSEHOLD HEAD HERE IS SAME AS HOUSEHOLD HEAD LISTED ON IDENTIFICATION PAGE.)


1.	2.	3.	4.	5.	6.		
MARK 'X' IF 	 Can you	Have you	What was the main reason you never	 What is the highest grade you	Are you		
THE MEMBER 	 read and	 ever	attended school?	 completed?	currently		
IS 7 YEARS OR	 write in any	attended		 	attending		
OLDER	 language?	school?	 1 = Working (Job) 	 1 = Initial  	school?		
			 2 = Working (home)	 2 = Primarily			
			 3 = Parents do not think it is important	 3 = High school 			
			 4 = Expensive	 4 = Preparatory school
  5 = TVET			
ONLY ASK			 5 = Too far away				
				
  6 = College 			
QUESTIONS IF			 6 = Marriage				
							
MEMBER IS 7			 7 = Illness/ disability	 7 = Degree 			
YEARS AND			 8 = Death of parent/s	 8 = MA/MSc.			
OLDER			 9 = Separation of parents	 9 = PhD			
			 10 = Other, specify.........................	 10 = Other, specify……..…………………			
	 1 = Yes
 2 = No


				1 = Yes 
2 = No


		
							
							
		1 = Yes >>Q-5
2 = No >>Q-4


					
							
							
							
							
							
							
							


          Section 3: Migration and Remittances 

         [Ask Household Head or Most Knowledgeable Member]

	1.	2.	3.	 4.	5. 	6.	7.	 8.		
I	 Please list all the former members 	 What is the [NAME's]	 What is the destination	Where does the	 Does  [NAME]	Indicate type of	 How many months 	 How many months		
N	 of the household who no longer 	 relationship to the 	 of that [NAME] does	[NAME] indicated 	 has legal 	migration?	 did [NAME] spent 	 did [NAME] spent		
D	 live with you for at least three 	 household head?	 not live here now?	 as an international	 residence in		 abroad?	 in local migration?		
I	 months. 			 migrant live in?	 this country?					
D			 1 = Domestic							
V	(THOSE WHO LEFT THE	 1 = Self, i.e.
       household head
 2 = Spouse
 
 3 = Child, step-               
        child
 4 = Parent
 5 = Other, specify………


	 2 = International >>Q-4			 				
I	FAMILY ONLY FOR LABOR										
D	MIGRANTION)					 1 = Yes					
U	 		 


	 1 = USA
 2 = UK
 3 = Canada
 4 = Australia
 5 = Germany 
 6 = Italy
 7 = Saudi Arabia 
 8 = Dubai 
 9 = Other,      
        specify…………	 2 = No	 1 = Temporary 				
A						 2 = Permanent 				
L										
										
I										
D										
										
										
										
										
										
										
										
										
										
										
										
								MONTHS	      MONTHS	
   11    											
   12											
   13 											
   14											
   15 											
   16											
   17 											
   18 											
   19											
   20											
        
	 9.	10.	11.	12.		13.		14.	15.	16.	17.	
I	 At the time [NAME]	 Do the [NAME's] 	Do [NAME] left	 Do [NAME] 	 Do [NAME] ever 	 Do [NAME] ever	 Did [NAME] 	 What job do [NAME]	 How much money	
N	 first moved to,	 current marital 	his/her spouse	 left his family 	 had a family or 	 had any ties with	 find work in 	 is currently working?	 did the [NAME] 		
D	 did s/he received any	 status is married?	 behind in the 	 for migration 	 friends who were 	 any organization	 the place where  		 make in the past 		
I	 help from family for		home country?	 intends to return	  there before s/he 	 in his current	 s/he is living?  	 1 = Professional	 12 months?		
V	 financing migration?			 home?		  left home?		 living area before		 2 = Sales, attendant… 			
D			 1 = Yes	 1 = Yes
 2 = No
 3 = Don't know
	1 = Yes		 s/he left home?
	 1 = Yes
 2 = No

 
 
 


	 3 = Operator, mechanic 
        fabricator….			
U			 									
		 	 2 = No		 2 = No				 4 = Private business     			
												
A	   1 = Yes	 1 = Yes >>Q-11	  						 5 = Housekeeper, 			
L	    2 = No	   2 = No     						 1 = Yes		 6 = Deriver, butcher, 
 7 = Daily laborer      
 8 = Other, specify…..
  
 


			
		  										
							 2 = No
					
I			  	 							
D		  	  									
													
											ETB		
													
   11    													
													
   12													
													
   13 													
													
   14													
													
   15 													
													
   16													
													
   17 													
   18 													
   19													
   20													


       Migration and Remittances


	18.	19.	20.	21.	22.	23.	24.	25.	 26.		
I	 Has [NAME] sent	  How frequently 	 How much money	Which means of	 What is the rate 	 Has [NAME] 	 Did [NAME] 	How much money 	 What was the main		
N	 any money to the	  does [NAME] 	 has [NAME] sent	money transferring	 of fee for sending 	 visited/returned 	 bring any money 	 in total did [NAME] 	 reasons which the		
D	 household over 		 send money?	 in total in the past	systems [NAME]   	 the money from 	 home during the	 with him/her 	 bring during the 	 migrant has sent		
I	 the past 12 			12 months?	used for sending 	 abroad?	  past 12 months?	 when s/he 	 past 12 months?	 money during the		
V	 months?				 the money?			  returned home?		 past 12 months? 		
I			 1 = Monthly	 DO NOT INCLUDE						 To finance…..		
D			 2 = Quarterly	 MONEY BROUGHT	  1 = Banks					  1 = Purchase of new teff
        variety 
  2 = Purchase of fertilizer
  3 = Purchase of    
       insecticides, herbicides 
  4 = Home consumption
  5 = Care of self-family
  6 = Purchase of land, 
        house, livestock…
  7 = Support education
  8 = Others, specify…


		
U			3 = Once/twice a year	 BACK IN PERSON 
 BY THE [NAME]	  2 = Postal service	  1 = None
  2 = Less than 1%
  3 = 1% - 3%
  4 = 3% - 5% 
   5 = More than 5%  


	 1 = Yes	 1 = Yes				
A			4 = Whenever needed	 	  3 = Relatives, friends		 2 = No	 2 = No				
L			 5 = Other, specify……	

	  4 = Other, MTOs		 					
I		  1   										
D	  1 = Yes >> Q-19	2										
	   2 = No 										
								  			
			ETB	  				     ETB			
			 International	 Domestic								
													
													
													
   11    													
   12													
   13 													
   14													
   15 													
   16													
   17 													
   18 													
   19													
   20													
        Migration and Remittances


Section 4B: Agriculture; Crop Production

[ASK HOUSEHOLDS THAT CULTIVATE ANY TPYE OF CROP]

	 1.		 2.	 3.	 4. 	 5.	 6.	 7.	 8.	 9. 	 10.	 11.	 12.	
	 Did you plant any of 	 What area did	 What type of	 If  improved, 	 What area 	 How much	 If hybrid, 	 What area	 
 How much	
 If conventional, 	
 What area	
 How much	
	 the following [CROP]	 you plant with	 seed did you 	 what was 	 did plant	 did you 	 what was 	 did plant	 did you spend 	 what was the 	 did plant	 did you  	
C	 during the last agrl		 the [CROP]?	 use for each 	 the quantity 	with	 spend on	 the quantity 	 with	 on hybrid 	 quantity of seed	 with 	 spend on 	
R	season?		 [CROP]?	 of seed 	 improved	 Improved	 of seed used 	 hybrid 	 seed for each 	 used for each	 conventional	 conventional	
O	 		 
 1 = Improved 
       >>Q-4
 2 = Hybrid 
       >>Q-7
 3 = Conventional 
       >>Q-10
 4 = More than 
       two types


	 used for 	 seed for	 seed for	 for each  	 seed for	 [CROP]?	 [CROP]? 	 seed for	 seed for each	
					 each 	 each 	 each 	 [CROP]? 	 each		                	 each	 [CROP]?	
P				 [CROP]? 	 [CROP]?	 [CROP]?	 	 [CROP]?	 	 	  [CROP]?		
													
C				 									
O	                 		  											
D	      1 = Yes >>Q-2													
E	      2 = No													
														
			 											
			    											
	 CROP	CODE	     HECTARE		KG	HECTARE	    ETB	KG	HECTARE	ETB	KG	HECTARE	ETB	
1	 Teff    													
2	 Maize													
3	 Wheat													
4	Barley 													
5	 Millet 													
6	Sorghum 													
7	 Pulses 													
8	Vegetables, 
 fruits  													
9	Oil seeds 													
10	Others crops 													


	 13.	 14.	 15. 	 16.	 17. 	 18. 	 19.	 20. 	 21. 	 22.  	 23.		
	 Did you use	 What quantity	 
 What was the	
 Did you use any	
 What was 	
 Did you use 	
 How do you 	
 What was the 	
 Did you use 	
 What kind of	
 How much did 		
	 any fertilizer 	 of fertilizer	 cost you spend	 chemical inputs	 the cost you 	 any farm 	 obtained the	 total cost you	 any animals  	 draft animals 	 you spend for		
	 on [CROP] 	 did you use 	 on fertilizer for	 to prevent any	 spend on 	 machineries 	 farm machi- 	 spend to buy 	 Power for 	 did you use on 	 for renting,		
C	 during last 	 for each  	 each [CROP]?	 damages on the	 chemical 	 during the last 	 neries?	 or rent  farm	 ploughing,	 this [CROP]?	 buying of the		
R	 season?	 [CROP]?	 	 [CROP]?	 inputs for 	 agrl season on 		 machineries for 	 harvesting 	 	 draft animals		
O	 		 		 each 	 [CROP]?		 each [CROP]?	  on [CROP]?	 	 meant for		
													
P			 	 	 [CROP]?		 1 = Owned		  1 = Yes Q-18	 	 crop 		
							 						
C	 1 = Yes 			 1 = Yes 		 1 = Yes >>Q-19	 2 = Purchased		  2 = No	 1 = Purchased	 production?		
O	  2 = No			 2 = No		 2 = No 	 3 = Rented 			 2 = Rented  			
D							 4 = Other,			 3 = Owned 			
E										 4 = Other,  			
										       specify			
				       						    			
													
		KG	ETB		ETB			ETB			ETB		
1													
2													
3													
4													
5													
6													
7													
8													
9													
10													


		24.	 25. 		
 C			 For the last agricultural season, how many days did your household hire men, women 	
 R		 Did you hire any labor on any of  	 and children for each activities on [CROP] wise?	
  O		 the following activities during 		
 P	 	 the last agricultural season on 		
 C		 the given [CROP]?	IF NONE HIRED: RECORD 0 FOR NUMBER OF MEN/WOMEN/CHILDREN (AND LEAVE 	
 O			DAYS AND WAGE BLANK)IF PAYMENT IN KIND, ESTIMATE VALUE IN ETHIOPIAN BIRR	
  D		  MARK 'X' IF YOU HIRE A LABOR 	MEN	WOMEN	CHILDREN (<15 YEARS)	
                E	 MAIN	  FOR ALL THE  ACTIVITIES BEFORE 										
	 [CROP]	  GOING TO QUESTIONS 21 TO 22.				DAILY WAGE
				DAILY WAGE

			 	DAILY WAGE	
																
		Activity Code	 MARK 'X'	#MEN	#DAYS	#Hours	ETB	#WOMEN	#DAYS	#Hours	ETB	#CHILDREN	#DAYS	#Hours	ETB	
1	 Teff 	Act1		 												
		Act2														
		Act3 														
		Act4 														
		Act5 														
		Act6 														
		Act7 														
		Act8 														
2	 Maize 	Act1		 												
		Act2														
		Act3 														
		Act4 														
		Act5 														
		Act6 														
		Act7 														
		Act8 														
3	 Wheat 	Act1		 												
		Act2														
		Act3 														
		Act4 														
		Act5 														
		Act6 														
		Act7 														
		Act8 														
CODE: Act1 = Land preparation; Act2 = Planting; Act 3 = Weeding; Act4 = Fertilizing; Act5 = Pest management; Act6 = Irrigation activities; Act7 = Harvesting; Act8 = Other activities

		 MARK 'X' IF YOU HIRE A LABOR FOR 	MEN	WOMEN	CHILDREN (<15 YEARS)	Men	WOMEN	CHILDREN (>15)		
	 MAIN	 ALL THE  ACTIVITIES BEFORE GOING TO 																	
	 [CROP]	 NEXT COLUMN. 				Daily wage			 Daily wage 			 Daily wage								
		Activity Code	 MARK 'X'
 	#MEN	#DAYS	ETB	#WOMEN	#DAYS	ETB	#CHILDREN	#DAYS	ETB	#MEN	#DAYS	#WOMEN	#DAYS	#CHILDREN	#DAYS		
4	 Barley	Act1		 																
		Act2																		
		Act3 																		
		Act4 																		
		Act5 																		
		Act6 																		
		Act7 																		
		Act8 																		
5	  Sorghum	Act1		 															
		Act2																	
		Act3 																	
		Act4 																	
		Act5 																	
		Act6 																	
		Act7 																	
		Act8 																	
CODE: Act1 = Land preparation; Act2 = Planting; Act 3 = Weeding; Act4 = Fertilizing; Act5 = Pest management; Act6 = Irrigation activities; Act7 = Harvesting; Act8 = Other activities


Section 4C: Agriculture; Harvesting    

[ASK HOUSEHOLDS THAT CULTIVATE ANY TPYE OF CROP]

	1.		2.	3.	 4.	5.	6.	7.	8.		
	 Did you harvest any of the following	 What quantity did	 How many kilos of	 What price did you	 How much did you	
 How much of the 
 harvested [CROP]
 was consumed?	 How much of	 How much of		
	 crops during the last agricultural	 you harvest from 	 [CROP] did you	 get from the sale of   	 receive from the		 the harvested	 the harvested		
	 season?		 each [CROP] in the 	 sell during the last	 each [CROP]?	 [CROP] that		 [CROP] was used for	 [CROP] is still		
C			 last 12 months?	 12 months?		 you sold?		 animal feed?	 stored?		
R	 ASK ABOUT ALL CROPS LISTED									
O	 BEFORE GOING TO QUESTIONS 2									
											
P	 THROUGH 11										
											
C											
O											
D		1 = Yes									
E		2 = No									
											
			 								
			    								
	 CROP	CODE	      KG	    KG	  ETB/KG	  ETB/KG	KG	KG	    KG		
1	Improved Teff										
2	Hybrid Teff										
3	Conventional/local Teff										
4	 Wheat										
5	 Maize 										
6	Barley 										
7	 Millet 										
8	 Sorghum 										
9	 Pulses 										
10	 Vegetables and fruits  										
11	 Oil seeds 										
12	Others crops 										


      Section 4D: Agriculture; Livestock

       [ASK BEST INFORMED RESPONDENT]

	1.		2.	3.		4.	5.	6.			
L	Does anyone in this household	 How many [ANIMAL]   
 does your household
 currently own?

	 How many [ANIMAL] did	 How much did you	 How many new [ANIMAL] 	What is the amount of		
I	breed or own livestock?			 you sell during the last	 receive for the sale	 did you buy during the last	total money spend to		
V				 12 months?		 of [ANIMAL]?	 12 months?	buy new [ANIMAL]? 		
E	 ASK ABOUT ALL ANIMALS LISTED							
S	 BEFORE GOING TO QUESTIONS 2								
T	 THROUGH 6										
O											
C											
K											
C		1 = Yes									
O		2 = No									
D						TOTAL GAIN IN ETB					
											
E							      NUMBER	TOTAL SPEND
IN ETB		
										
	 LIVESTOCK	    CODE	NUMBER	NUMBER					
1	Milk cow							
2	Oxen 							
3	Beef cattle 							
4	Camel  							
5	Sheep, lamp 							
6	Goats 							
7	Poultry/all chickens  							
8	Horses 							
9	Donkey, mules 							
10
	Beehives 							
11	Other, specify							
          


Section 5: Access to Basic Services
1.	2.	3.	 4. 	 5.			6.		 7. 		
								
 Is there an extension 	 Are there trained 	 Did you get the  
 required extension 
 Information in the
   last 12 months?
  

 1 = Yes >>Q-4
 2 = No


	What kind of information did you get from 
 the extension agent's visit?

  


 1 = Utilization of improved teff
 2 = Advisory on plantation and sequences 
        of farm practices on improved teff
 3 = Application of fertilizer and chemical 
        inputs
 4 = Technical advisory on post-harvest
 5 = Input and output market information
 6 = Others, specify………..

	 How many contacts 	 Do you have a near place to buy	 Where is the possible place 
 to get  access to improved teff?


			
center nearest to you?	 extension agents 			 did you receive from	 improved teff variety anytime you				
	 permanently working			 the extension agent?	 want?			
 1 = Yes >>Q-2	 at this extension			 1 = Every day
 2 = Every week
 3 = Twice a month
  4 = Every month

	 
  1 = Yes >>Q-7 
  2 = No


			
	 center?							
	 1 = Always
      available
 2 = Sometimes
               available
 3 =  Never
       available

							
  2 = No								
						 1 = Governmental sectors  
 2 = Cooperative societies  
 3 = Private suppliers   
 4 = Regular market 
 5 = Other, specify 


		
								
								
								
 								
								
								
								
								
								
										
										
										
										
										
										
										
									
									
									
										
										
												
 8.	 9. 	 10.	11.	 12.	 13.		
								
 For how long did you use Improved teff seeds 
 on your farm? For at last…

 1 = One season
 2 = Two seasons
 3 = Three seasons
 4 = More than three
       seasons


	 How do you rate the   
 intensity of adoption	 Did you faced pests and diseases on this
 improved seed variety during the last	 Did you get pest management help
 to prevent the damage on teff crop?


 1 = Yes >>Q-12
 2 = No


				
	 of improved teff	 agricultural season?					
	 seed at a defined 						
	 level of distribution?						
		        1 = Yes >>Q-11					
	  1 = High	        2 = No					
		 						
		 						
								
	  2 = Moderate							
	 3 = Low

							
								

 				
				
								
								
								
          

        Section 6: Annual Income and Household Assets 


[ASK BEST INFORMED RESPONDENTS]                                                                           

	 Annual Income           	  	 Household Assets        	
	 1.		 2.	  	 1.	 2. 	 3. 	 4.	
  S	 Did anyone in this household received	 How much income		Did anyone in this household	How many of the 	How did you obtained 	If you wanted to sell the 	
  O	 income from any of the following 	 did you get from		possesses any of the following  	[ITEM] does the 	the [ITEM]? 	[ITEM], how much 	
  U	 [SOURCE] during the last 12 months?	 the [SOURCE]?	I	[ITEM]?


	household own? 		would you be able to	
  R	 		 	T				 sell it for today?	
  C			E				 	
  			M			 1 = Purchase >>Q-4		
							  2 = Gift/aid 		
				C			     3 = Given as payment		
  C				O			 4 = Other, specify		
  O				D					
  D				E	                                    1 = Yes				
  E					                                    2 = No				
		1 = Yes							
		2 = No							
									
	ITEM	CODE	TOTAL ETB		ITEM	 CODE	NUMBER		ETB	
					Households asset 					
1	Crop sales			1	Color TV					
2	Vegetables and fruit sales			2	Black TV					
3	Livestock sales			3	Video player, DVD					
4	Sales of livestock products 			4	Camera 					
5	Vegetables and fruit sales			5	Mobile phone					
6	Gifts from relatives, friends			6	Satellite antenna 					
7	Aids from institutions 			7	Audio system 					
8	Off-farm income 			8	Stove 					
9	Sales of assets 			9	Refrigerator 					
10	Remittances and transfers			10	Bicycle 					
11	Rental income			11	Sofa set 					
12	Other sources of income			12	Wrist watch					
					Farm tools					
				13	Generator/Pump					
				14	Cart (hand pushed) 					
				15	Cart (animal drawn)					
				16	Other, specify 					


Section 7: Natural Resource Conservation 
1.	2.	3.	 4. 	 5.			6.		 7. 		
								
 Does any of the HH   
 member undertake   
 any natural resource 
 conservation     
 measures?

 1 = Yes >>Q-2
  2 = No
 	 Planting trees or        
 hedges on household 
 farmland and/or 
 forested land? 

 1 = Yes 
  2 = No


	 Protecting trees in  
 household farmland 
 and/or forested land?  


 1 = Yes 
 2 = No


	 Building stone or soil ridges on sloping 
 farmland to prevent soil erosion?
  


 1 = Yes 
 2 = No
	 Mending terrace 
 ridges to prevent soil  
 erosion ?


 1 = Yes 
 2 = No
	 Maintaining and improving  
  irrigation of farmland?


  1 = Yes 
  2 = No


	 Converting sloping farmland  
 into terraces? 


 
  1 = Yes 
  2 = No

		
								
								
								
								
								
								
						 


		
								
								
								
								
								
								
								
								
								
								
								
								
								
								
								
								
								
								
								
										

8.	9.	10.	 11. 	 12.			13.		 		
								
 Increasing the   
 use of organic farm  
 fertilizer? 


 1 = Yes 
  2 = No
 	 Reducing the use of 
 artificial fertilizer and  
 other chemicals? 


 1 = Yes 
  2 = No


	 Planting legumes and 
 other kinds of green 
 manure crops?  


 1 = Yes 
 2 = No


	 Practicing fallowing?
  


 1 = Yes 
 2 = No
	 Manually weeding 
 household farmland 
 and/or forested land ?

 1 = Yes 
 2 = No
	 Acquiring information on natural 
 resources and the environment 
 from sources such as television, 
 newspapers, and magazines?


  1 = Yes 
  2 = No


	 		
								
								
								
								
								
								
						 


		
								
								
								
								
								
								
								
								
								
								
								
								
								
								
								
								
								
								
								
										


THE END
~~~~~~//~~~~~~~


SECTION 8: CONTACT INFORMATION

1. In order for us to be able to contact the household in the future, could you kindly provide us with telephone numbers?

PHONE NUMBER FOR HOUSEHOLD HEAD:	LANDLINE	CELL	
	NAME : _____________________________________	PHONE: ___________________________/_______________________________	


2. In case we are not able to make contact with the household head, could you kindly provide us with the telephone numbers of some other adult members of this household ?

PHONE NUMBERS FOR OTHER HOUSEHOLD MEMBERS:

     A.	NAME : _____________________________________	ID (FROM ROSTER)	__________	PHONE  : ___________________________________	
    B.	NAME : _____________________________________	ID (FROM ROSTER)	__________	PHONE : ___________________________________	
    C.	NAME : _____________________________________	ID (FROM ROSTER)	__________	PHONE : ___________________________________	
